# Could an Optimized Joint Pharmacokinetic/Pharmacodynamic Target Attainment of Continuous Infusion Piperacillin-Tazobactam Be a Valuable Innovative Approach for Maximizing the Effectiveness of Monotherapy Even in the Treatment of Critically Ill Patients with Documented Extended-Spectrum Beta-Lactamase-Producing *Enterobacterales* Bloodstream Infections and/or Ventilator-Associated Pneumonia?

**DOI:** 10.3390/antibiotics12121736

**Published:** 2023-12-14

**Authors:** Milo Gatti, Matteo Rinaldi, Tommaso Tonetti, Antonio Siniscalchi, Pierluigi Viale, Federico Pea

**Affiliations:** 1 Department of Medical and Surgical Sciences, Alma Mater Studiorum University of Bologna, 40138 Bologna, Italy; milo.gatti2@unibo.it (M.G.); mat.rinaldi1989@gmail.com (M.R.); tommaso.tonetti@unibo.it (T.T.); pierluigi.viale@unibo.it (P.V.); 2 Clinical Pharmacology Unit, Department for Integrated Infectious Risk Management, IRCCS Azienda Ospedaliero-Universitaria of Bologna, 40138 Bologna, Italy; 3 Infectious Disease Unit, Department for integrated Infectious Risk Management, IRCCS Azienda Ospedaliero-Universitaria of Bologna, 40138 Bologna, Italy; 4 Division of Anesthesiology, Department of Anesthesia and Intensive Care, IRCCS Azienda Ospedaliero-Universitaria di Bologna, 40138 Bologna, Italy; 5 Anesthesia and Intensive Care Medicine, IRCCS Azienda Ospedaliero-Universitaria di Bologna, 40138 Bologna, Italy; antonio.siniscalchi@aosp.bo.it

**Keywords:** piperacillin-tazobactam, continuous infusion, critically ill patients, Gram-negative infections, joint PK/PD target attainment, expert clinical pharmacological advice, microbiological eradication

## Abstract

(1) Background: Piperacillin-tazobactam represents the first-line option for treating infections caused by full- or multi-susceptible *Enterobacterales* and/or *Pseudomonas aeruginosa* in critically ill patients. Several studies reported that attaining aggressive pharmacokinetic/pharmacodynamic (PK/PD) targets with beta-lactams is associated with an improved microbiological/clinical outcome. We aimed to assess the relationship between the joint PK/PD target attainment of continuous infusion (CI) piperacillin-tazobactam and the microbiological/clinical outcome of documented Gram-negative bloodstream infections (BSI) and/or ventilator-associated pneumonia (VAP) of critically ill patients treated with CI piperacillin-tazobactam monotherapy. (2) Methods: Critically ill patients admitted to the general and post-transplant intensive care unit in the period July 2021–September 2023 treated with CI piperacillin-tazobactam monotherapy optimized by means of a real-time therapeutic drug monitoring (TDM)-guided expert clinical pharmacological advice (ECPA) program for documented Gram-negative BSIs and/or VAP were retrospectively retrieved. Steady-state plasma concentrations (C_ss_) of piperacillin and of tazobactam were measured, and the free fractions (*f*) were calculated according to respective plasma protein binding. The joint PK/PD target was defined as optimal whenever both the piperacillin *f*C_ss_/MIC ratio was >4 and the tazobactam *f*C_ss_/target concentration (C_T_) ratio was > 1 (quasi-optimal or suboptimal whenever only one or none of the two weas achieved, respectively). Multivariate logistic regression analysis was performed for testing variables potentially associated with microbiological outcome. (3) Results: Overall, 43 critically ill patients (median age 69 years; male 58.1%; median SOFA score at baseline 8) treated with CI piperacillin-tazobactam monotherapy were included. Optimal joint PK/PD target was attained in 36 cases (83.7%). At multivariate analysis, optimal attaining of joint PK/PD target was protective against microbiological failure (OR 0.03; 95%CI 0.003–0.27; *p* = 0.002), whereas quasi-optimal/suboptimal emerged as the only independent predictor of microbiological failure (OR 37.2; 95%CI 3.66–377.86; *p* = 0.002). (4) Conclusion: Optimized joint PK/PD target attainment of CI piperacillin-tazobactam could represent a valuable strategy for maximizing microbiological outcome in critically ill patients with documented Gram-negative BSI and/or VAP, even when sustained by extended-spectrum beta-lactamase (ESBL)-producing *Enterobacterales*. In this scenario, implementing a real-time TDM-guided ECPA program may be helpful in preventing failure in attaining optimal joint PK/PD targets among critically ill patients. Larger prospective studies are warranted to confirm our findings.

## 1. Introduction

Sepsis and septic shock are major causes of patient admission in intensive care units (ICU) worldwide [1,2,3]. Additionally, they may also represent common complications occurring in patients previously admitted in the ICU for other underlying diseases [1,2,3]. Sepsis is characterized by high morbidity and mortality, and the most common infectious agent causing it is bacteria, so it is associated with conspicuous antibiotic consumption [2,4]. Specifically, pneumonia and bloodstream infections (BSIs) represent the most frequent cause of sepsis in ICU patients, and Gram-negative pathogens, namely *Enterobacterales* and *Pseudomonas aeruginosa*, are responsible for a large proportion of cases [3,5,6].

According to several recent guidelines and opinion articles, piperacillin-tazobactam is considered a first-line option for treating infections caused by full- or multi-susceptible *Enterobacterales* and/or *Pseudomonas aeruginosa* [7,8,9,10]. Additionally, piperacillin-tazobactam could also be considered as a carbapenem-sparing alternative for managing infections caused by extended-spectrum beta-lactamase (ESBL)-producing *Enterobacterales,* even in critically ill patients [9,11,12]. 

Several clinical studies showed that attaining an aggressive pharmacokinetic/pharmacodynamic (PK/PD) target of 100%*f*T_>4–8 × MIC_ with continuous infusion (CI) beta-lactams among critically ill patients was associated with both the maximization of clinical efficacy and the suppression of resistance development [13,14,15,16,17,18,19,20]. Failure in early attainment of this aggressive PK/PD target of piperacillin-tazobactam was shown to be as high as 80% during intermittent or extended infusion administration [21], and around 5–28% during CI administration [22,23,24,25,26]. All studies assessing aggressive PK/PD target attainment of piperacillin-tazobactam were based solely on piperacillin concentrations, without taking tazobactam exposure into account. Indeed, with regard to beta lactam (BL)–beta lactamase inhibitor (BLI) combinations, including piperacillin-tazobactam, the innovative concept of the so-called joint PK/PD target was recently proposed [27,28], in which optimal PK/PD target attainment should be achieved for both the BL and for the combined BLI simultaneously [27]. Additionally, it should not be overlooked that selecting the most appropriate dosing regimen may be extremely challenging in critically ill patients, considering that piperacillin-tazobactam pharmacokinetics may be significantly affected by sepsis-related pathophysiological changes [29,30,31,32]. Consequently, implementing a real-time therapeutic drug monitoring (TDM)-based expert clinical pharmacological advice (ECPA) program could play a relevant role in optimizing promptly the joint PK/PD target attainment of antimicrobials, including piperacillin-tazobactam, among critically ill patients [22]. 

The aim of this study was to assess the relationship between the joint PK/PD target attainment of CI piperacillin-tazobactam and the microbiological/clinical outcome of documented Gram-negative BSIs and/or ventilator-associated pneumonia (VAP) of critically ill patients treated with piperacillin-tazobactam monotherapy. 

## 2. Results

**Figure 1:**
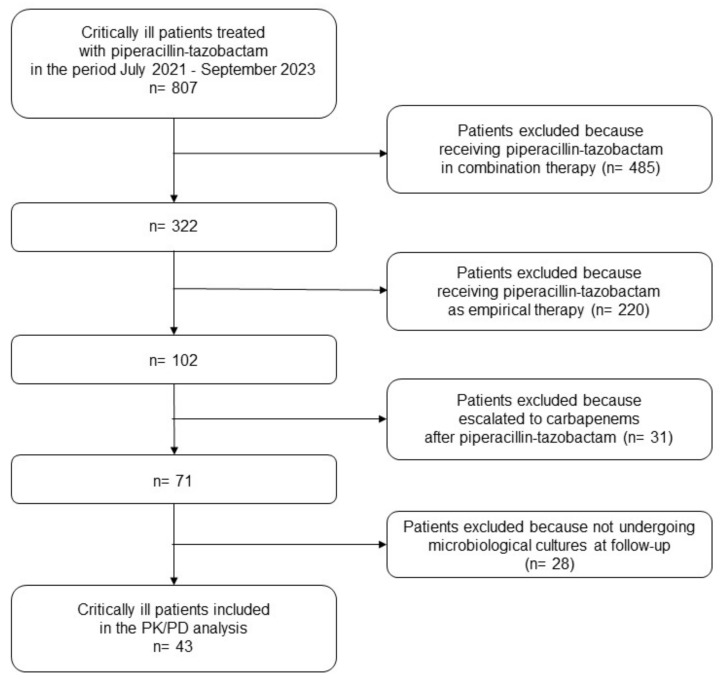
Flowchart of patient inclusion and exclusion criteria. PK/PD: pharmacokinetic/pharmacodynamic analysis.

Overall, a total of 807 critically ill patients admitted to the general ICU and to the post-transplant ICU were treated with CI piperacillin-tazobactam during the study period. Among these, 43 underwent a TDM-based ECPA approach for personalizing CI piperacillin-tazobactam monotherapy of documented Gram-negative BSIs and/or VAP, and were included in the PK/PD analysis (Figure 1). Demographics and clinical features of the patients are reported in Table 1. 

Median (interquartile range (IQR)) age was 69 years (57–64 years), with a slight male preponderance (58.1%). Post-anoxic coma after resuscitated cardiac arrest and bowel occlusion/perforation (5 cases each; 11.6%) were the most common underlying diseases. 

Median (IQR) Sequential Organ Failure Assessment (SOFA) score at baseline was 8 (4–11). Thirty-five patients (81.4%) underwent invasive mechanical ventilation, and 27 (62.8%) required cardiovascular support with vasopressors. Continuous renal replacement therapy (CRRT) was applied in 11 cases (25.6%), and augmented renal clearance occurred in 3 cases (7.0%). 

BSIs, VAP and bacteremic VAP were documented in 24 (55.8%), 16 (37.2%) and 3 (7.0%) patients, respectively. Overall, 48 different Gram-negative pathogens were isolated, with *Escherichia coli* (18 cases; 37.5%), *Pseudomonas aeruginosa* (14 cases; 29.0%) and *Klebsiella pneumoniae* (6 cases; 12.5%) being the most common ones. Fully susceptible and beta-lactamase producing pathogens accounted for 79.2% (38/48) and for 20.8% (10/48) of clinical isolates, respectively. Specifically, among beta-lactamase producing pathogens, ESBL-producing and AmpC-producing *Enterobacterales* accounted for 14.6% (7/48) and for 6.2% (3/48) of clinical isolates, respectively.

Piperacillin-tazobactam was administered at a median (IQR) daily dose of 18 g (13.5 g-18 g) for a median (IQR) of 9 days (7–12 days). Overall, 93 TDM-based ECPAs for optimizing CI piperacillin-tazobactam exposure were performed, with a median (IQR) of 2 (1–2.5) assessments per patient. Median (IQR) piperacillin and tazobactam free steady-state concentrations (*f*C_ss_) were 54.6 mg/L (41.0–91.2 mg/L) and 7.2 mg/L (4.6–11.6 mg/L), respectively. The median piperacillin *f*C_ss_/minimum inhibitory concentration (MIC) ratio and avibactam *f*C_ss_/target concentration (C_T_) ratio were 7.6 (4.8–13.0) and 1.8 (1.2–2.9), respectively. Dosing adjustments at first TDM-based ECPA were recommended in 28 out of 43 patients (65.1%), with one increase (2.3%) and 27 decreases (62.8%). Overall, dosing adjustments were recommended in 44 out of 93 TDM-based ECPAs (47.3%), with five increases (5.4%) and 39 decreases (41.9%). The joint PK/PD target of piperacillin-tazobactam was optimal in 36 patients (83.7%), quasi-optimal in 6 (14.0%) and suboptimal in one (2.3%).

Microbiological eradication was documented in 32 patients (74.4%), clinical cure was achieved in 29 patients (67.4%), and resistance to piperacillin-tazobactam occurred in 3 patients (7.0%). Four patients (9.3%) were colonized at 90-day by multidrug-resistant (MDR) Gram-negative pathogens. Median (IQR) delta SOFA score at day 2 and at day 7 were 0 (0–2) and 2 (0–4.5), respectively. ICU and 30-day mortality rate were 9.3% and 14.0%, respectively.

Univariate and multivariate regression analysis of variables associated with patients having optimal vs. quasi-optimal/suboptimal joint PK/PD target attainment of piperacillin-tazobactam is summarized in Table 2.

At univariate analysis, optimal joint PK/PD target attainment of piperacillin-tazobactam was significantly associated both with a higher microbiological eradication rate (86.1% vs. 14.3%; *p* < 0.001) and with a higher clinical cure rate (77.8% vs. 14.3%; *p* = 0.003), and had a trend towards a lower ARC occurrence (2.8% vs. 27.8%; *p* = 0.06) and a higher need for vasopressors (69.4% vs. 28.6%; *p* = 0.08) compared to quasi-optimal/suboptimal joint PK/PD target attainment. No significant difference between the two groups was found in terms of resistance occurrence (5.6% vs. 14.3%; *p* = 0.42), novel colonization with MDR Gram-negatives (8.3% vs. 14.3%; *p* = 0.52), delta SOFA score at 48 h (0 vs. 2; *p* = 0.37) and at day 7 (2.5 vs. 1; *p* = 0.64) and ICU mortality rate (11.1% vs. 0.0%; *p* = 0.99). At multivariate analysis, optimal PK/PD target attainment was confirmed as being a significant predictor of microbiological eradication (odds ratio (OR) 0.03; 95% confidence interval (CI) 0.003–0.27; *p* = 0.002).

Univariate and multivariate analyses assessing variables associated with microbiological eradication vs. microbiological failure are shown in Table 3.

**Figure 2:**
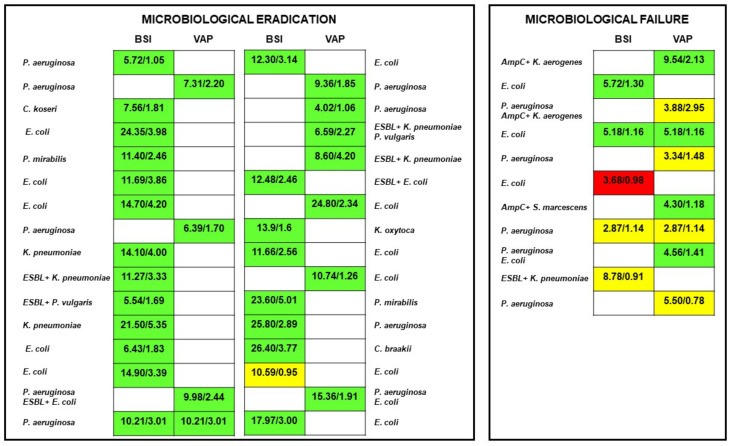
Relationship between microbiological outcome and optimal (green box), quasi-optimal (yellow box) or suboptimal (red box) joint PK/PD target attainment of piperacillin-tazobactam. Microbiological failure rate was significantly higher among patients attaining quasi-optimal/suboptimal joint PK/PD target of piperacillin-tazobactam than in those attaining optimal target (54.5% vs. 2.9%; *p* < 0.001). BSI: bloodstream infections; VAP: ventilator-associated pneumonia.

At multivariate analysis, quasi-optimal/suboptimal joint PK/PD target attainment of piperacillin-tazobactam emerged as the only independent predictor of microbiological failure (odds ratio (OR) 37.2; 95% confidence interval (CI) 3.66–377.86; *p* = 0.002; Figure 2), whereas ARC, VAP, and infections caused by AmpC-producing pathogens were not retained in the final model.

## 3. Discussion

To the best of our knowledge, this is the first study to explore the relationship between the joint PK/PD target attainment of CI piperacillin-tazobactam monotherapy and the microbiological/clinical outcome of documented Gram-negative BSI and/or VAP in critically ill patients treated with piperacillin-tazobactam monotherapy. The findings showed that real-time TDM-guided ECPA of CI piperacillin-tazobactam enabled optimal joint PK/PD target attainment in more than 80% of critically ill patients. Failure in attaining this target was the sole independent predictor of microbiological failure of CI piperacillin-tazobactam monotherapy in the treatment of critically ill patients with documented Gram-negative BSI and/or VAP.

Critically ill patients attaining optimal joint PK/PD target of CI piperacillin-tazobactam had significantly higher microbiological eradication than those attaining only quasi-optimal/suboptimal target. The findings are consistent with those of several recent studies showing that aggressive PK/PD target attainment with beta-lactams, defined as 100%*f*T_>4–8×MIC_, was associated with better microbiological and/or clinical outcome [16,17,18,19,20,28,33,34]. 

However, our study goes also beyond by proposing an innovative PK/PD approach for maximizing the effectiveness of piperacillin-tazobactam. With regard to BL/BLIc, we first introduced the concept of joint PK/PD target attainment, pointing out that, when using an BL/BLIc, it is important to consider an optimized PK/PD target attainment not only of the BL but also of the BLI [28]. We believe that attaining optimal joint PK/PD target may be especially worthwhile when dealing with piperacillin-tazobactam monotherapy against very severe and challenging microbiological/clinical conditions. In this regard, some preclinical studies may support this contention by showing that increasing tazobactam concentrations may result in a consistent MIC decrease in piperacillin when dealing with infections with high inocula [35,36], such as VAP and/or with ESBL-producing *Enterobacterales* [35,37,38]. 

Optimal joint PK/PD target attainment of piperacillin-tazobactam was found to be protective against microbiological failure, irrespective of the infection site, even if a trend toward higher microbiological failure rate was observed for VAP or bacteremic VAP compared to BSI. In this regard, it may be speculated that the vast majority of VAP patients experiencing microbiological failure attained only borderline values of optimal joint PK/PD target in plasma. Considering that piperacillin showed limited penetration rate in the epithelial lining fluid of critically ill patients [39,40], this could have resulted in only quasi-optimal/suboptimal joint PK/PD target attainment at the infection site. Consequently, for overcoming this issue, it may be prudent when dealing with VAP adopting a more restrictive PK/PD target of piperacillin, namely *f*C_ss_/MIC ratio of 6–8 rather than 4–8, as just previously suggested in a recent opinion article [22]. 

Regarding infections sustained by beta-lactamase-producing bacteria, it is worth noting that microbiological eradication occurred in most of the cases caused by ESBL-producing *Enterobacterales* (6/7; 85.7%; 4 BSI and 2 VAP), all attaining optimal joint PK/PD target, but in none of those caused by AmpC-producing *Enterobacterales* (3/3; 0%; 3 VAP). In the Merino trial, the 30-day mortality rate of BSIs caused by ESBL-producing bacteria was higher in the piperacillin-tazobactam arm than in the meropenem arm (12.3 vs. 3.7%). However, it should not be overlooked that inadequate piperacillin-tazobactam exposure related to intermittent infusion administration was suggested as a factor potentially contributing to this difference [41]. Consequently, it may be speculated that an optimized joint PK/PD target attainment of CI piperacillin-tazobactam could be a valuable and innovative approach for maximizing the effectiveness of monotherapy, even for the treatment of critically ill patients with documented ESBL-producing *Enterobacterales* BSI and/or VAP. Conversely, failure in eradicating AmpC-producing *Enterobacterales* regardless of optimized joint PK/PD target attainment may reiterate once more the contention that piperacillin-tazobactam should not be considered a valuable agent against these pathogens.

Quasi-optimal/suboptimal joint PK/PD target of piperacillin-tazobactam had a trend toward higher occurrence among patients with ARC and/or not needing vasopressor support. This may be related to the faster renal elimination of piperacillin-tazobactam occurring under these circumstances [42], and is in agreement with several studies showing that ARC and/or no need for vasopressor support may be significant predictors of failure in attaining optimal PK/PD targets with beta-lactams, possibly leading to a worse clinical outcome [29,33,34,43,44,45,46,47,48,49,50,51]. In this challenging scenario, implementing a real-time TDM-guided ECPA program could be helpful in promptly identifying critically ill patients at high risk of attaining only quasi-optimal/suboptimal joint PK/PD target of piperacillin-tazobactam, thus favoring better clinical and microbiological outcome compared to standard approaches [52,53]. 

Limitations of our study should be recognized. The retrospective monocentric study design and the limited sample size must be acknowledged. Total piperacillin and tazobactam concentrations were measured, and the free fractions were only estimated. Conversely, the high homogeneity of our cohort, composed of critically ill patients receiving only monotherapy with piperacillin-tazobactam for treating documented Gram-negative infections, allowed us to avoid any confounding factor in assessing the relationship between PK/PD target attainment and microbiological/clinical outcome.

## 4. Materials and Methods

### 4.1. Study Design and Inclusion Criteria

Critically ill patients admitted to the general ICU and to the post-transplant ICU of the IRCCS Azienda Ospedaliero-Universitaria of Bologna, Italy in the period between 1 July 2021 and 15 September 2023 were retrospectively screened. Patients were included if: (a) they had a documented BSIs or pneumonia caused by Gram-negative pathogens with available MIC value for piperacillin-tazobactam and received CI piperacillin-tazobactam monotherapy for at least 48 h during ICU stay; (b) they underwent optimization of piperacillin-tazobactam exposure according to a real-time TDM-guided ECPA program with at least one TDM assessment available performed during ICU stay; (c) they had no escalation or de-escalation of therapy during the piperacillin-tazobactam treatment course; (d) they did not die or have had compassionate care in the first 48 h after ICU admission. 

Monotherapy was defined as the absence of use of any concomitant antimicrobials, including antibiotic, antifungal or antiviral agents. Piperacillin-tazobactam TDM-guided ECPA assessments performed outside of ICU stay were excluded from the analysis. 

### 4.2. Data Collection and Variables Definition

For each included critical patient, demographic data (age, sex, weight, height, body mass index (BMI)); clinical/laboratory data (underlying disease leading to ICU admission, requirement for mechanical ventilation and/or for vasopressors, CRRT application, CLC_R_ at baseline, and occurrence of ARC); microbiological data (type/site of infection, isolated Gram-negative pathogens with relative MIC values); antibiotic treatment data (piperacillin-tazobactam dosing at baseline, steady-state concentrations (C_ss_) of both piperacillin and tazobactam at first TDM-guided ECPA; average piperacillin and tazobactam C_ss_ during treatment course in patients underwent more than one TDM-guided ECPA; overall number of ECPAs; recommended dosing adjustments at first and at subsequent ECPAs, treatment duration); and outcome data (microbiological eradication, resistance development, clinical cure, delta 48-h SOFA and delta 7-days SOFA, MDR colonization at 90-day, ICU and 30-day mortality rate) were retrieved. 

ARC was defined as a measured (based on 24 h urine collection) or estimated (according to the CDK-EPI formula) creatinine clearance above 130 mL/min and 120 mL/min in males and females, respectively [54].

The Centers for Disease Control and Prevention (CDC) criteria were adopted for defining the different types of infection [55]. Specifically, isolation of a Gram-negative pathogen from blood cultures identified a documented BSI [55], whereas detection of a bacterial load ≥10^4^ CFU/mL of one or more Gram-negative isolates in the bronchoalveolar lavage (BAL) fluid culture collected at least 48 h after endotracheal intubation, coupled with new or progressive lung infiltrates identified a documented VAP [56,57].

A semi-automated broth microdilution method (Microscan Beckman NMDRM1) was adopted for determining piperacillin-tazobactam susceptibility against Gram-negative *Enterobacterales* and *Pseudomonas aeruginosa*. The European Committee on Antimicrobial Susceptibility Testing (EUCAST) clinical breakpoints were adopted for interpreting the MIC values [58]. Threshold values of ≤8 mg/L and ≤16 mg/L identified *Enterobacterales* and *Pseudomonas aeruginosa* isolates susceptible to piperacillin-tazobactam [58,59].

Absence of the index Gram-negative pathogen from the primary site of infection in at least two subsequent assessments denoted effective microbiological eradication, whereas the persistence of the index Gram-negative isolate at follow-up cultures executed at least seven days after starting piperacillin-tazobactam treatment course pointed out microbiological failure [60]. 

An increase in piperacillin-tazobactam MIC values above the EUCAST susceptibility clinical breakpoint denoted resistance development.

Clinical cure was assumed to be achieved whenever the complete resolution of signs and symptoms of the infectious disease was coupled with documented microbiological eradication and absence of any recurrence or relapse at 30-day follow-up [61]. 

The SOFA score was calculated at baseline (defined as the day of starting piperacillin-tazobactam therapy), at 48 h and at day 7. The delta 48-h SOFA score was considered as the difference between SOFA score calculated at baseline and at 48 h, whereas the delta 7-day SOFA score was calculated as the difference between the SOFA score at baseline and that calculated at day 7. For critically ill patients who were discharged from the ICU or who died before day 7, the last available SOFA score value was taken into account for calculating the delta 7-day SOFA score. 

MDR colonization was defined as the detection of a novel difficult-to-treat resistant (DTR) pathogen in surveillance rectal swabs without any sign or symptom of infection in the 90 days after starting piperacillin-tazobactam therapy.

### 4.3. Piperacillin-Tazobactam Dosing Regimens, Sampling Procedure, and Procedure for Optimizing PK/PD Target Attainment

Piperacillin-tazobactam was started with a loading dose of 9 g administered over 2 h infusion, followed by an initial maintenance dose administered by CI over 24 h, thanks to stability in aqueous solution [62]. Initial maintenance dose regimen was defined on a case-by-case basis according to pathophysiological conditions of each patient, renal function and site of infection. 

Total piperacillin and tazobactam plasma concentrations were first measured at least 24 h after starting treatment by means of a validated liquid chromatography-tandem mass spectrometry method [19]. TDM-guided ECPA reassessments were performed, if needed, every 48–72 h during ICU stay. To obtain *f* piperacillin and tazobactam C_ss_, the total C_ss_ values were multiplied by 0.80 and 0.77 values, respectively, based on the plasma protein binding rates retrieved in the literature [63].

A real-time TDM-guided ECPA program was implemented for optimizing piperacillin-tazobactam exposure. Specifically, piperacillin-tazobactam TDM results were interpreted by an MD Clinical Pharmacologist with long-standing expertise in optimizing antimicrobial therapy in critically ill patients, by providing a dedicated ECPA [22]. Furthermore, MD Clinical Pharmacologists attended Monday-to-Friday the morning bedside multidisciplinary meeting in the ICUs.

### 4.4. Definition of Optimized Joint PK/PD Target Attainment of Piperacillin-Tazobactam

The so-called joint PK/PD target was selected as best PD determinant of piperacillin-tazobactam efficacy. Specifically, the joint PK/PD target was defined as optimal when both the piperacillin *f*C_ss_/MIC ratio was >4 and the tazobactam *f*C_ss_/C_T_ ratio was >1 (where C_T_ corresponded to the fixed tazobactam target concentration used by the EUCAST for the in vitro standard susceptibility testing, namely, 4 mg/L), and quasi-optimal or suboptimal when only one or none of the two thresholds were attained, respectively [27]. Attainment of this aggressive PK/PD target with beta-lactams was previously associated with both maximal clinical efficacy and suppression of resistance emergence among Gram-negative pathogens [13,14,15,16,17,18,19]. Piperacillin-tazobactam dosing adjustments were performed whenever needed, as previously reported [22].

Average piperacillin and tazobactam *f*C_ss_ were calculated in patients undergoing multiple TDM-guided ECPAs during the overall treatment course. The relationships existing between the joint PK/PD target attainment of piperacillin-tazobactam and the clinical/microbiological outcomes (in terms of microbiological eradication, resistance development, clinical cure, delta 48-h and 7-days SOFA, acquisition of novel MDR colonization and mortality) were assessed.

### 4.5. Statistical Analysis

Continuous data were expressed as median and interquartile range (IQR), and categorical variables were presented as counts or percentages. Univariate analyses between patients attaining optimal vs. quasi-optimal/suboptimal piperacillin-tazobactam joint PK/PD target, and between patients with microbiological eradication vs. microbiological failure were carried out by applying the Fisher’s exact test or the chi-squared test for categorical variables, or the Mann–Whitney U test for continuous variables. Multivariate logistic regression analysis was carried out for identifying independent predictors associated with microbiological failure. All the variables resulted significant at the univariate analysis (*p* value < 0.05) were included in the multivariate logistic regression model. *p* values < 0.05 defined statistical significance. Statistical analyses were carried out by means of the MedCalc statistical software (Version 19.6.1, Ostend, Belgium).

## 5. Conclusions

Overall, our findings suggest that an optimized joint PK/PD target attainment of CI piperacillin-tazobactam could represent a valuable strategy for maximizing microbiological outcome in critically ill patients with documented Gram-negative BSI and/or VAP, even when sustained by ESBL-producing *Enterobacterales*. In this scenario, implementing a real-time TDM-guided ECPA program may be helpful in preventing failure in attaining optimal joint PK/PD targets among critically ill patients. Larger prospective studies are warranted to confirm our findings. 

## Figures and Tables

**Table 1:** Demographics and clinical characteristics of included ICU patients receiving CI piperacillin-tazobactam for documented Gram-negative BSIs and/or VAP.

Demographics and Clinical Variables	ICU Patients (N = 43)
*Patient demographics*	
Age (years) (median (IQR))	69 (57–74)
Gender (male/female) (n (%))	25/18 (58.1/41.9)
Body weight (Kg) (median (IQR))	80 (65–90)
Body mass index (Kg/m^2^) (median (IQR))	26.1 (23.1–29.4)
*Underlying diseases* (n (%))	
Post-anoxic coma after resuscitated cardiac arrest	5 (11.6)
Bowel occlusion/perforation	5 (11.6)
Acute pulmonary edema	4 (9.3)
Solid cancer	4 (9.3)
Drug intoxication	4 (9.3)
Acute pancreatitis	2 (4.7)
OLT	2 (4.7)
ARDS in COVID-19	2 (4.7)
Inflammatory bowel disease	2 (4.7)
Other ^a^	13 (30.1)
*Severity of clinical conditions*	
Baseline SOFA score (median (IQR))	8 (4–11)
Mechanical ventilation (n (%))	35 (81.4)
Vasopressors (n (%))	27 (62.8)
Baseline CL_CR_ (mL/min/1.73 m^2^) (median (IQR))	88.0 (57.3–102.0)
Continuous renal replacement therapy (n (%))	11 (25.6)
Augmented renal clearance (n (%))	3 (7.0)
*Site of infection* (n (%))	
BSI	24 (55.8)
VAP	16 (37.2)
VAP + BSI	3 (7.0)
*Gram-negative clinical isolates* ^b^ (n (%))	
*Escherichia coli*	18 (37.5)
*Pseudomonas aeruginosa*	14 (29.0)
*Klebsiella pneumoniae*	6 (12.5)
*Klebsiella aerogenes*	2 (4.2)
*Proteus mirabilis*	2 (4.2)
*Proteus vulgaris*	2 (4.2)
*Serratia marcescens*	1 (2.1)
*Citrobacter koseri*	1 (2.1)
*Citrobacter braakii*	1 (2.1)
*Klebsiella oxytoca*	1 (2.1)
*Piperacillin-tazobactam treatment*	
Daily dose (mg) (median (IQR))	18 g/day (13.5–18 g/day)
Treatment duration (days) (median (IQR))	9 (7–12)
Piperacillin *f*C_ss_ (mg/L) (median (IQR))	54.6 (41.0–91.2)
Tazobactam *f*C_ss_ (mg/L) (median (IQR))	7.2 (4.6–11.6)
Piperacillin *f*C_ss_/MIC ratio (median (IQR))	7.6 (4.8–13.0)
Tazobactam *f*C_ss_/C_T_ ratio )median (IQR)]	1.8 (1.2–2.9)
*PK/PD target attainment*	
Overall optimal joint PK/PD target (n (%))	36 (83.7)
Overall quasi-optimal joint PK/PD target (n (%))	6 (14.0)
Overall suboptimal joint PK/PD target (n (%))	1 (2.3)
*ECPA program*	
Overall TDM-based ECPAs	93
N. of TDM-based ECPA per treatment course (median (IQR))	2 (1–2.5)
N. of dosage confirmations at first TDM assessment (n (%))	15 (34.9)
N. of dosage increases at first TDM assessment (n (%))	1 (2.3)
N. of dosage decreases at first TDM assessment (n (%))	27 (62.8)
Overall n. of dosage confirmations (n (%))	49 (52.7)
Overall n. of dosage increases (n (%))	39 (41.9)
Overall n. of dosage decreases (n (%))	5 (5.4)
*Outcome*	
Microbiological eradication (n (%))	32 (74.4)
Resistance occurrence (n (%))	3 (7.0)
Clinical cure (n (%))	29 (67.4)
90-days MDR colonization (n (%))	4 (9.3)
Delta 48-h SOFA (median (IQR))	0 (0–2)
Delta 7-days SOFA (median (IQR))	2 (0–4.5)
ICU mortality (n (%))	4 (9.3)
30-day mortality (n (%))	6 (14.0)

ARDS: acute respiratory distress syndrome; BSI: bloodstream infection; CL_CR_: creatinine clearance; ECPA: expert clinical pharmacological advice; *f*C_ss_: free steady-state concentrations; *f*C_T_: free threshold concentrations; ICU: intensive care unit; IQR: interquartile range; MDR: multidrug-resistant; MIC: minimum inhibitory concentration; OLT: orthotopic liver transplant; PK/PD: pharmacokinetic/pharmacodynamic; SOFA: sequential organ failure assessment; TDM: therapeutic drug monitoring; VAP: ventilator-associated pneumonia. ^a^ acute kidney injury (N = 1); urinary lithiasis (N = 1); non-ST segment elevation myocardial infarction (N = 1); Guillain–Barre syndrome (N = 1); acute-on-chronic liver failure (N = 1); hemorrhagic shock (N = 1); polytrauma (N = 1); acute respiratory insufficiency (N = 1); chronic obstructive pulmonary disease (N = 1); myasthenia gravis (N = 1); abdominal wall hematoma (N = 1); coma of unknow origin (N = 1); thrombotic thrombocytopenic purpura (N = 1). ^b^ Overall, 48 different Gram-negative pathogens were identified in the 43 ICU patients.

**Table 2:** Univariate and multivariate analysis of variables associated with patients having optimal vs. quasi-optimal/suboptimal joint PK/PD target attainment of piperacillin-tazobactam.

Variables	Optimal Joint PK/PD Target Attainment (N = 36)	Quasi-Optimal/Suboptimal Joint PK/PD Target Attainment (N = 7)	Univariate Analysis *p* Value	Multivariate Analysis (OR; 95%CI)	Multivariate Analysis *p* Value
*Patient demographics*					
Age (years) (median (IQR))	68.5 (56.75–73.5)	69 (63.5–73)	0.79		
Gender (male/female) (n (%))	19/17 (52.8/47.2)	6/1 (85.7/14.3)	0.21		
Body weight (Kg) (median (IQR))	75 (65–90)	81 (77.5–102.5)	0.18		
Body mass index (Kg/m^2^) (median (IQR))	26.0 (22.8–28.5)	31.3 (26.3–32.5)	0.11		
*Severity of clinical conditions*					
Baseline SOFA score (median (IQR))	8.5 (5.75–11)	4 (3–11)	0.38		
Mechanical ventilation (n (%))	28 (77.8)	7 (100.0)	0.31		
Vasopressors (n (%))	25 (69.4)	2 (28.6)	0.08		
Continuous renal replacement therapy (n (%))	10 (27.8)	1 (14.3)	0.66		
Augmented renal clearance (n (%))	1 (2.8)	2 (27.8)	0.06		
*Site of infection* (n (%))					
BSI	21 (58.3)	3 (42.9)	0.68		
VAP	13 (36.1)	3 (42.9)	0.99		
VAP + BSI	2 (5.6)	1 (14.2)	0.42		
*Outcome*					
Microbiological eradication (n (%)])	31 (86.1)	1 (14.3)	**<0.001**	0.03 (0.003–0.27)	**0.002**
Resistance occurrence (n (%))	2 (5.6)	1 (14.3)	0.42		
Clinical cure (n (%))	28 (77.8)	1 (14.3)	**0.003**	-	
90-day MDR colonization	3 (8.3)	1 (14.3)	0.52		
Delta 48-h SOFA score (median (IQR))	0 (0–2)	2 (0–3)	0.37		
Delta day 7 SOFA score (median (IQR))	2.5 (0–5)	1 (0–3)	0.64		
ICU mortality (n (%))	4 (11.1)	0 (0.0)	0.99		
30-day mortality (n (%))	6 (16.7)	0 (0.0)	0.57		

BSI: bloodstream infection; CI: confidence interval; ICU: intensive care unit; IQR: interquartile range; MDR: multidrug-resistant; OR: odds ratio SOFA: sequential organ failure assessment; VAP: ventilator-associated pneumonia.

**Table 3:** Univariate and multivariate analysis comparing patients with microbiological eradication vs. microbiological failure.

Variables	Microbiological Eradication (N = 32)	Microbiological Failure (N = 11)	Univariate Analysis *p* Value	Multivariate Analysis (OR; 95%CI)	Multivariate Analysis *p* Value
*Patient demographics*					
Age (years) (median (IQR))	68.5 (56.75–76.25)	69 (59–70.5)	0.79		
Gender (male/female) (n (%))	18/14 (56.3/43.7)	7/4 (63.6/36.4)	0.74		
Body weight (Kg) (median (IQR))	75 (64.25–90)	81 (72.5–92.5)	0.32		
Body mass index (Kg/m^2^) (median (IQR))	25.7 (22.4–28.9)	27.8 (26.0–30.3)	0.16		
*Severity of clinical conditions*					
Baseline SOFA score (median (IQR))	8 (5.75–11)	9 (3–11)	0.54		
Mechanical ventilation (n (%))	24 (75.0)	11 (100.0)	0.09		
Vasopressors (n (%))	21 (65.6)	6 (54.5)	0.72		
Continuous renal replacement therapy (n (%))	9 (28.1)	2 (18.2)	0.70		
Augmented renal clearance (n (%))	0 (0.0)	3 (27.3)	**0.01**	-	
*Site of infection* (n (%))					
VAP or bacteremic VAP	11 (34.4)	8 (72.7)	**0.04**	-	
*Gram-negative clinical isolates* (n (%))					
*Escherichia coli*	15 (42.8)	3 (23.0)	0.32		
*Pseudomonas aeruginosa*	9 (25.7)	5 (38.5)	0.48		
*Klebsiella pneumoniae*	4 (11.4)	2 (15.4)	0.66		
*Klebsiella aerogenes*	0 (0.0)	2 (15.4)	0.07		
*Proteus mirabilis*	2 (5.7)	0 (0.0)	0.99		
*Proteus vulgaris*	2 (5.7)	0 (0.0)	0.99		
*Serratia marcescens*	0 (0.0)	1 (7.7)	0.27		
*Citrobacter koseri*	1 (2.9)	0 (0.0)	0.99		
*Citrobacter braakii*	1 (2.9)	0 (0.0)	0.99		
*Klebsiella oxytoca*	1 (2.9)	0 (0.0)	0.99		
*Susceptibility pattern*					
Full-susceptible	26 (82.9)	7 (69.2)	0.25		
ESBL-producing *Enterobacterales*	6 (17.1)	1 (7.7)	0.66		
AmpC-producing *Enterobacterales*	0 (0.0)	3 (23.1)	**0.01**	-	
*Piperacillin-tazobactam treatment and joint PK/PD target attainment*					
Quasi-optimal/suboptimal joint PK/PD target attainment	1 (2.9)	6 (54.5)	**<0.001**	37.2 (3.66–377.86)	**0.002**

IQR: interquartile range; OR: odds ratio; PK/PD: pharmacokinetic/pharmacodynamic; SOFA: sequential organ failure assessment; VAP: ventilator-associated pneumonia.

## Data Availability

The data presented in this study are available on request from the corresponding author. The data are not publicly available due to privacy concerns.
